# First Report on a Cluster of Colistin-Resistant *Klebsiella pneumoniae* Strains Isolated from a Tertiary Care Center in India: Whole-Genome Shotgun Sequencing

**DOI:** 10.1128/genomeA.01466-16

**Published:** 2017-02-02

**Authors:** Purva Mathur, Balaji Veeraraghavan, Naveen Kumar Devanga Ragupathi, Francis Yesurajan Inbanathan, Surbhi Khurana, Nidhi Bhardwaj, Subodh Kumar, Sushma Sagar, Amit Gupta

**Affiliations:** a Department of Laboratory Medicine, All India Institute of Medical Sciences, New Delhi, India; b Department of Clinical Microbiology, Christian Medical College, Vellore, India

## Abstract

*Klebsiella pneumoniae* is a nosocomial pathogen with clinical importance due to its increasing resistance to carbapenems and colistin. Here, we report the genome sequences of eight colistin-resistant *K. pneumoniae* strains which might help in understanding the molecular mechanism of the species. The sequence data indicate genomes of ~5.2 to 5.4 Mb, along with several plasmids.

## GENOME ANNOUNCEMENT

*Klebsiella pneumoniae* is gaining importance due to increased carbapenem and colistin resistance with very few treatment options (1). Resistance to colistin is mediated by chromosomal mutations and plasmids (2). This study investigates the antimicrobial resistance mechanisms of a cluster of carbapenem-resistant *K. pneumoniae* isolates with special reference to colistin.

In this study, we present the draft genome sequences of eight *Klebsiella pneumoniae* isolates (PM565, PM1842, PM1995, PM138, PM716, PM1134, PM5186, and PM1168) from clinical samples. The isolates were observed to be phenotypically resistant to meropenem and colistin by the *E*-test method.

To further understand the mechanism behind colistin resistance, whole-genome sequencing was performed using the Ion Torrent PGM platform with 400-bp chemistry. *De novo* assembly of raw data was achieved using AssemblerSPAdes version 5.0.0.0 embedded in the Torrent suite server version 5.0.3. Sequence annotation was done in PATRIC, the bacterial bioinformatics database and analysis resource (http://www.patricbrc.org) (3), the Rapid Annotations using Subsystems Technology (RAST) server (http://rast.nmpdr.org) (4, 5), and the NCBI Prokaryotic Genome Annotation Pipeline (PGAP, http://www.ncbi.nlm.nih.gov/genomes/static/Pipeline.html).

Complete details about the eight genomes are given in Table 1. The assembly resulted in 105 to 160 contigs (≥500 bp). The genome coverages of these isolates were about 32× to 51×. The genomes had coding sequences ranging from 5,859 to 6,744; rRNAs from 11 to 14; and tRNAs from 64 to 73. The annotation revealed 27, 26, 29, 34, 38, 36, 36, and 35 antimicrobial-resistance genes from the ARDB database and 77, 75, 82, 89, 96, 97, 91, and 84 from the CARD database for the genomes of isolates B565, UI842, B1995, BAL138, B716, B1134, P5186, and B1168, respectively. Similarly, for the virulence genes, the VFDB and Victors database revealed the presence of 81, 82, 89, 106, 69, 67, 85, and 82, and 177, 177, 184, 188, 187, 185, 187, and 180 genes, respectively.

**TABLE 1:** Whole-genome shotgun sequence details of colistin-resistant *Klebsiella pneumoniae* isolates

Isolate	Genome size (bp)	Genome coverage (×)	No. of contigs	Coding sequences; rRNAs; tRNAs	Sequence type	ResFinder	PlasmidFinder	Accession no.
PM565	5,650,616	40	127	5,945; 14; 69	ST-11	*blaOXA-232*, *blaTEM-1B*, *blaCTX-M-15*, *blaSHV-11*, *aacA4*, *strA*, *strB*, *rmtf*, *aac(6′)Ib-cr*, *qnrB1*, *fosA*, *ARR-2*, *sul2*	ColKP3, IncR, IncFII(K), IncHI1B, IncFIB(pQil)	MNPB00000000
PM1842	5,577,703	43	110	5,859; 14; 70	ST-11	*blaOXA-232*, *blaTEM-1B*, *blaCTX-M-15*, *blaSHV-11*, *aacA4*,* strA*, *strB*, *rmtf*, *aac(6′)Ib-cr*, *qnrB1*, *fosA*, *ARR-2*, *sul2*	ColKP3, IncR, IncFII(K), IncHI1B, IncFIB(pQil)	MNPC00000000
PM1995	5,583,954	51	105	6,056; 13; 70	ST-11	*blaTEM-1B*, *blaCTXM-15*, *blaLEN12*, *aacA4*, *strA*, *strB*, *rmtf*, *aac(6′)Ib-cr*, *qnrB1*, *fosA*, *ARR-2*, *sul2*	IncR, IncFII(K), IncHI1B, IncFIB(pQil)	MNPD00000000
PM138	5,774,696	38	160	6,744; 13; 66	ST-11	*blaOXA-232, blaTEM-1B*, *blaCTXM-15*, *blaSHV-11*, *aacA4*,* strA*, *strB*, *rmtf*, *aac(6′)Ib-cr*, *qnrB1*, *fosA*, *ARR-2*, *sul2*	ColKP3, IncR, IncFII(K), IncHI1B, IncFIB(pQil)	MNPG00000000
PM716	5,727,677	43	108	6,415; 13; 69	ST-14	*blaOXA-232*, *blaTEM-1B*, *blaOXA-9*, *blaOXA-1*, *blaSHV-28*, *blaNDM-1*, *blaCTX-M-15*, *aadA2*, *aadA1*, *aacA4*, *armA*, *aph(3′)-via*, *aac(6′)Ib-cr*, *qnrS1*, *oqxA*, *oqxB*, *fosA*, *msr(E)*, *Mph(E)*, *catB3*, *sul1*,* dfrA12*, *dfrA1*	IncHI1B, IncFIB(mar), IncFIB(pQil), IncFII(K), ColKP3	MNPH00000000
PM1134	5,735,750	40	132	6,615; 12; 64	ST-14	*blaOXA-181*, *blaTEM-1B*, *blaOXA-9*, *blaOXA-1*, *blaSHV-28*,* blaNDM-1*, *blaCTX-M-15*, *aadA2*, *aadA1*, *aacA4*, *armA*, *aph(3′)-via*,* aac(6′)Ib-cr*, *qnrS1*, *oqxA*, *oqxB*, *fosA*, *msr(E)*,* Mph(E)*, *catB3*, *sul1*,* dfrA12*, *dfrA1*	IncHI1B, IncFIB(mar), IncFIB(pQil), IncFII(K), ColKP3	MNPF00000000
PM5186	5,440,451	41	111	6,194; 12; 73	ST-231	*blaTEM-1B*, *blaSHV-1*, *blaCTX-M-15*, *aacA4, rmtf*, *aadA2*,* aac(6′)Ib-cr*, *oqxA*, *oqxB*, *fosA*, *mph(A)*, *erm(B)*, *catA1*, *ARR-2*,* sul1*, *dfrA12*	IncFIB(pQil), IncFII(K), IncFIA	MNPE00000000
PM1168	5,554,499	32	119	5,907; 11; 68	ST-231	*blaOXA-232*, *blaTEM-1B*, *blaSHV-12*, *blaCTXM-15*, *aadA2*,* rmtf*, *aacA4*, *aac(6′)Ib-cr*, *qnrS1*, *oqxA*, *oqxB*, *fosA*, *mph(A)*,* erm(B)*, *catA1*, *ARR-2*, *sul1*, *dfrA12*	IncFIB(pQil), IncFII(K), IncFIA	MNPA00000000

Moreover, the sequence types of the isolates were found to be ST-11 for PM565, PM1842, PM1995, and PM138; ST-14 for PM716 and PM1134; and ST-231 for PM5186 and PM1168, as analyzed by the MLST version 1.8 tool (https://cge.cbs.dtu.dk//services/MLST) (6). ResFinder version 2.1 (http://www.cbs.dtu.dk/services) returned multiple antimicrobial resistance genes for most of the antibiotic classes (Table 1). Interestingly, fosfomycin-, fluoroquinolone-, aminoglycoside-, and β-lactam-resistant determinants were found in all eight isolates. However, the plasmid-mediated colistin-resistance determinants *mcr*1 and *mcr*2 were not found in any of the isolates. Analysis of the plasmids using PlasmidFinder version 1.3 (http://www.cbs.dtu.dk/services) identified IncFII(K) and IncFIB(pQil) in all isolates in addition to a few other plasmids (Table 1).

### Accession number(s).

The whole-genome sequences of the eight *K. pneumoniae* isolates were deposited in GenBank/DDBJ under the accession numbers mentioned in Table 1.
